# Heterogeneity correction for intensity-modulated frameless SRS in pituitary and cavernous sinus tumors: a retrospective study

**DOI:** 10.1186/s13014-015-0500-y

**Published:** 2015-09-17

**Authors:** Lisa B. E. Shields, Cindy Bond, Aaron Odom, David A. Sun, Aaron C. Spalding

**Affiliations:** Norton Neuroscience Institute, Norton Healthcare, 210 East Gray Street, Suite 1102, Louisville, KY 40202 USA; The Norton Cancer Institute Radiation Center, Louisville, KY USA; The Brain Tumor Center, Norton Healthcare, Louisville, KY USA; Associates in Medical Physics, LLC, Greenbelt, MD USA

**Keywords:** Radiation, Oncology, Radiosurgery, Dosimetry, Pituitary tumor, Cavernous sinus tumor

## Abstract

**Background:**

Frameless immobilization allows for planning and quality assurance of intensity-modulated radiosurgery (IM-SRS) plans. We tested the hypothesis that IM-SRS planning with uniform tissue density corrections results in dose inaccuracy compared to heterogeneity-corrected algorithms.

**Methods:**

Fifteen patients with tumors of the pituitary or cavernous sinus underwent frameless IM-SRS. Treatment planning CT and MRI scans were obtained and fused to delineate the tumor, optic nerves, chiasm, and brainstem. The plan was developed with static gantry IM-SRS fields using a pencil beam (PB), analytical anisotropic (AAA), and Acuros XB (AXB) algorithms. We evaluated measures of target coverage as well as doses to organs at risk (OAR) for each algorithm. We compared the results of each algorithm in the cases where PTV overlapped OAR (*n* = 10) to cases without overlapping OAR with PTV (*n* = 5). Utilizing film dosimetry, we measured the dose distribution for each algorithm through a uniform density target to a rando phantom with non-uniform density of air, tissue, and bone.

**Results:**

There was no difference in target coverage measured by D_MaxPTV_, D_MinPTV_, D_95%PTV_, or the isodose surface (IDS) covering 95 % of the PTV regardless of algorithm. However, there were differences in dose to OAR. PB predicted higher (*p* < 0.05) Dmax for the brainstem, chiasm, right optic nerve, and left optic nerve. In cases of PTV overlapping an optic nerve (*n* = 7), PB was unable to limit dose to 8Gy while achieving PTV coverage (PB 855 cGy vs. AAA 769 cGy, *p* = 0.05 vs. AXB 658 cGy, *p* = 0.03). Within the rando phantom, the PB and AAA algorithms over-estimated the dose delivered in the bone-tissue-air interface of the sinus (+17 %), while the AXB algorithm closely predicted the actual dose delivered through the inhomogeneous tissue (+/- 1 % max, *p* < 0.05).

**Conclusions:**

Patients undergoing frameless SRS benefit from heterogeneity corrected dose plans when the lesion lies in areas of widely varying tissue density and near critical normal structures such as the skull base. Film dosimetry confirms that the AXB dose calculation algorithm more accurately predicts actual dose delivered though tissues of varying densities than PB or AAA dose calculation algorithms.

## Introduction

Radiosurgery plays an important role in the treatment of both benign and malignant intracranial neoplasms [1, 2]. Linear accelerator (LINAC) radiosurgery has proven efficacious in utilizing a frame-based concept of treating these tumors [3, 4]. The patient is required to wear a rigid head frame to ensure immobilization and positioning, which limits the time allowed for radiation and planning quality assurance (QA). A frameless device has been developed for intracranial radiosurgery which avoids patient discomfort associated with the rigid head frame [5, 6]. It has been proven to have clinical feasibility and accuracy compared to frame-based systems when used in conjunction with image guidance [6, 7]. Frameless immobilization allows for planning and quality assurance of complicated intensity-modulated radiosurgery (IM-SRS) plans, which would be predicted to improve target dose while minimizing critical tissue doses.

Dose gradients and tissue inhomogeneity effects should be considered during the entire process of intensity-modulated radiotherapy (IMRT) planning [8]. Lesions near the pituitary fossa and cavernous sinus benefit from IM-SRS because the generated steep dose gradient allows tumoricidal doses while sparing the optic nerves and chiasm. Treatment of this location, however, poses IM-SRS planning difficulties due to the variation of electron density ranging from bone to tissue to air. Failure to accurately account for the tissue heterogeneity could result in dose inaccuracies leading to either toxicity or decreased tumor control.

In the traditional radiosurgery environment where solitary cranial metastases are treated with cones, conformal beams, or dynamic conformal arcs, the issue of tissue inhomogeneity is mostly irrelevant due to the uniformity of the cranial tissue inside the skull. In this uniform environment the robustness of tissue corrections becomes irrelevant and the extended calculation times which accompany the complexity of the algorithms employing them become unnecessary. For this reason, fast and simple algorithms which use somewhat rudimentary heterogeneity corrections, or no such corrections at all, have continued to have relevance in cranial radiosurgery treatment planning.

The pencil beam (PB) algorithm is the primary method utilized in routine clinical treatment planning for proton radiotherapy as it is reasonably accurate and fast [8, 9]. It has not been validated for use in highly inhomogeneous areas. PB algorithms have long been in place for all types of radiation therapy treatment planning. The Analytical Anisotropic Algorithm (AAA) is a convolution-superposition-based photon-beam dose computation algorithm which is fast and more accurate for IMRT than PB [10, 11]. More recently, complex algorithms employing robust corrections for tissue inhomogeneities have become available. These algorithms have become widely used in many treatment applications, yet the simpler algorithms have persisted in the radiosurgery environment. Acuros XB (AXB) is a photon dose calculation algorithm and is the most recently developed. Of the three algorithms, AXB is the most valid and accurate when applied to inhomogeneous media [12].

Radiation therapy dose calculation algorithms typically compute dose to water. Recent algorithms, like AXB (Varian Medical Systems, Palo Alto, CA) which attempt to improve accuracy in dose calculations in heterogeneous media, offer either absorbed dose to water [D_(w)_] or absorbed dose to the material [D_(m)_] [13]. Neglecting to take into account differences between D_(m)_ and D_(w)_ in a heterogeneous environment will lead to systematic dose errors [13]. Differences between these two methods have been reported to exceed 10 % for electron beams [14], and the dose to the bone was larger by up to 10 % when calculating D_(w)_ instead of D_(m)_ for phantoms and head and neck patients [14].

We tested the hypothesis that frameless IM-SRS planning in pituitary and cavernous sinus tumors with uniform tissue density corrections results in dose inaccuracy compared to algorithms accounting for tissue heterogeneity. The accuracy of the divergent heterogeneity corrections was evaluated with film dosimetry. This manuscript analyzes the consequences of using different calculation algorithms for the final dose calculation of a modulated treatment plan optimized based on fluence calculation with PB. Because we found differences between the three calculation algorithms, we measured the dose deposited in a phantom modeling real treatment conditions.

## Methods

A total of 15 patients with tumors near air/bone/brain interfaces, specifically, of the pituitary or cavernous sinus underwent frameless IM-SRS. The institutional IRB approved the retrospective study of these cases. Treatment planning CT (0.6 mm slices) and MRI (1.0 mm slices) scans were obtained and fused in order to delineate the tumor as well as the optic nerves, chiasm, and brainstem by the treating radiation oncologist and neurosurgeon. The initial plan was developed with static gantry IM-SRS fields, and volume dose was calculated using a PB algorithm (D_(w)_). Volume dose was then recalculated with identical monitor units and mlc movement patterns with AAA (D_(w)_) and AXB (D_(m)_) algorithms. We evaluated measures of target coverage as well as doses to OAR for each algorithm. We also compared the results of each algorithm in the cases where PTV overlapped OAR (*n* = 10) and when there were no overlapping OAR with PTV (*n* = 5).

Utilizing film dosimetry, we measured the accuracy of the predicted dose distribution for each algorithm in solid water as well as through tissue to bone to air within a rando phantom. Using a NovalisTX linear accelerator, a single 6MV photon beam delivering 12Gy was exposed through gafchromicXB2 film on a solid water phantom and through the sinus of a rando phantom.

Commissioning each algorithm requires independent sets of data for the purpose of beam modeling. The authors addressed the uncertainty introduced by the differences in collecting and processing of the base data by separating the film dosimetry validation into two steps. The uniformity and simplified geometry of the solid water phantom was used to verify the congruence of the dose prediction between algorithms without the complications of heterogeneity [15]. A film was placed between slabs of solid water and arranged perpendicular to the beam incidence. This test was designed to demonstrate consistency of data collection and commissioning between algorithms. Blank films were subtracted from the irradiated film with consideration to the orientation of the film on the scanner. A film calibration file was applied to convert optical density to dose. The film was compared to dose planes and profiles predicted by each of the algorithms, and consistency in the beam data between algorithms was confirmed.

We compared the predicted measures of dose to the PTV and OAR using a two-tailed Student’s *t*-test between PB and AAA or AXB as well as the measured film dose in the rando phantom between the algorithms.

## Results

### All cases

**Fig. 1:**
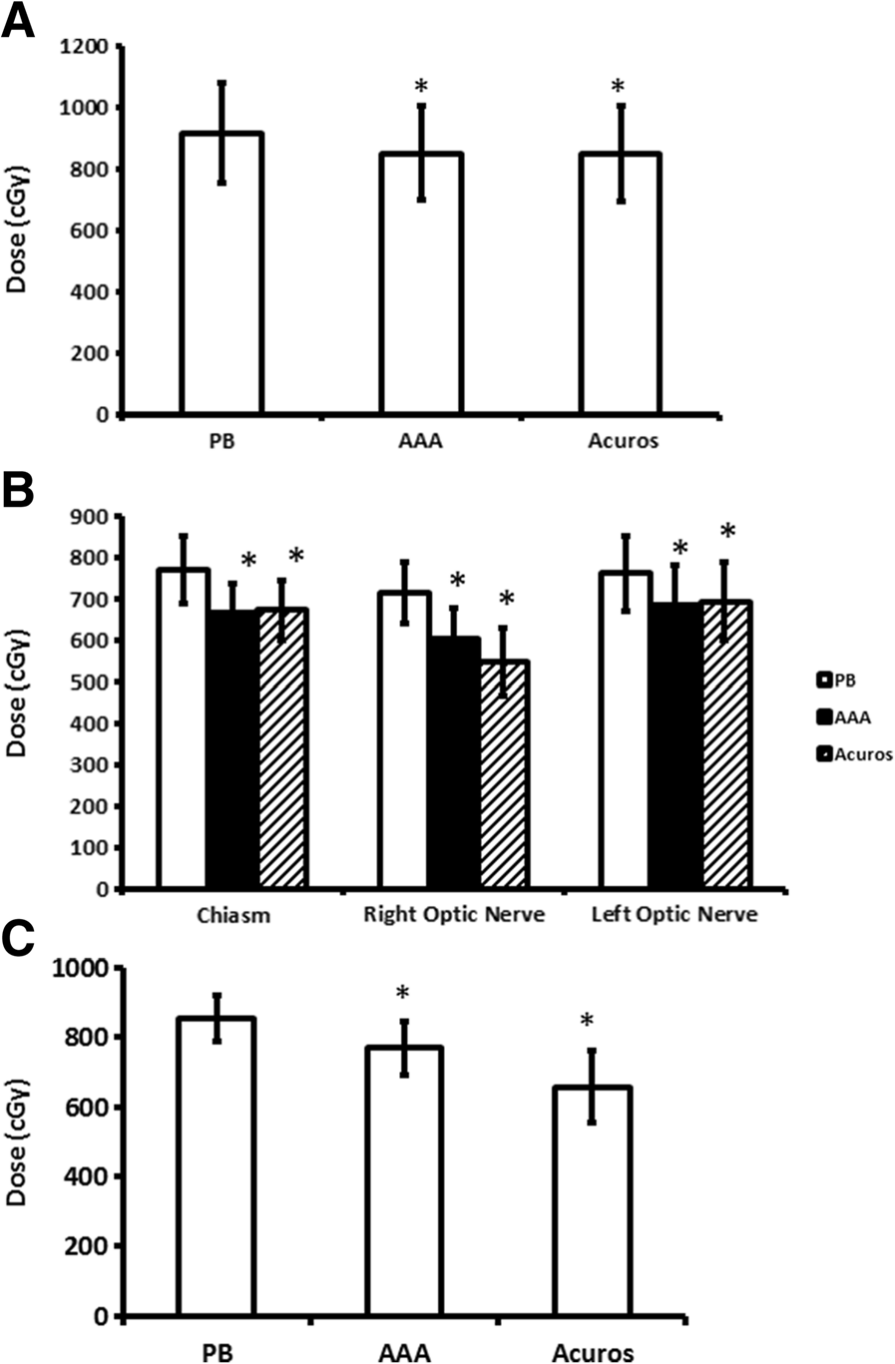
**a** Maximum brainstem dose for all cases (*n* = 15). **b** Maximum chiasm and optic nerve doses for single fraction SRS cases (*n* = 10). **c** Maximum optic nerve dose in cases with PTV overlap (*n* = 7). * indicates *p* < 0.05 for all figures between PB and the designated algorithm

The initial analysis consisted of both single and five fraction cases (*n* = 15). We found there was no difference in target coverage between PB, AAA, or AXB. There was no difference in D_MaxPTV_ between PB (115 % ± 14 %), AAA (113 % ± 13 %), or AXB (116 % ± 14 %). In addition, D_MinPTV_ was no different for each algorithm (PB [73 % ± 20 %], AAA [71 % ± 21 %], AXB [73 % ± 19 %]), and D_95%PTV_ was no different for each algorithm (PB [94 % ± 12 %], AAA [93 % ± 11 %], AXB [92 % ± 12 %]). The IDS covering 95 % of the PTV was no different for each algorithm (PB [81 % ± 35 %, AAA [93 % ± 11 %], AXB [83 % ± 34 %]). The maximum brainstem dose was higher for PB (916 ± 685 cGy) compared to AAA (850 ± 645 cGy, p = 0.004 vs PB) and AXB (851 ± 659 cGy, p = 0.002 vs PB) (Fig. 1a).

### Single fraction radiosurgery cases

The analysis was then limited to single fraction cases (*n* = 10) as the accepted maximum dose to the chiasm and optic nerves was 8 Gy [16]. For these cases, the pass rate was evaluated relative to the 8Gy tolerance dose to the optic nerves. Calculations predicting maximum doses below 8Gy to both optic nerves were considered passing with respect to this criterion. As in other areas of this study, optimized fluence was held constant and volume dose was compared between PB, AAA, and AXB with pass rates of 40, 50, and 60 % respectively.

PB predicted a statistically significant higher maximum dose than either AAA or AXB to the chiasm (771 ± 255 cGy vs 666 ± 228 cGy [*p* = 0.0004 vs PB] vs 693 ± 229 cGy [*p* = 0.001 vs PB]); the right optic nerve (715 ± 231 cGy vs 602 ± 233 [*p* = 0.01 vs PB] vs 547 ± 255 cGy [*p* = 0.01 vs PB]); and left optic nerve (762 ± 288 cGy vs 685 ± 305 cGy [*p* = 0.02 vs PB] vs 673 ± 299 cGy [*p* = 0.01 vs PB]) (Fig. 1b).

### Organs at risk

In order to determine the influence of heterogeneity correction in the areas of maximum dose gradients, we examined the effect of PB versus AAA and AXB when PTV and OAR overlapped (*n* = 10) compared to the cases without overlap (*n* = 5). For the 10 cases with overlap, there were 22 total overlapping structures, ranging from 1-3 per case. The right optic nerve overlapped in all ten cases, while the left optic nerve and chiasm overlapped in six cases, respectively. Since the maximum tolerated dose was well established for single fraction radiosurgery, we compared the maximum doses to the optic nerve for the overlap (*n* = 7) and non-overlapping (*n* = 4) cases that received a single fraction. In cases of PTV overlapping an optic nerve (*n* = 7), PB calculations resulted in higher doses predicted while achieving PTV coverage (PB 855 ± 179 cGy vs. AAA 769 ± 203 cGy, p = 0.05 vs. AXB 658 ± 270 cGy, p = 0.03) (Fig. 1c).

## Phantom film measurements

**Fig. 2:**
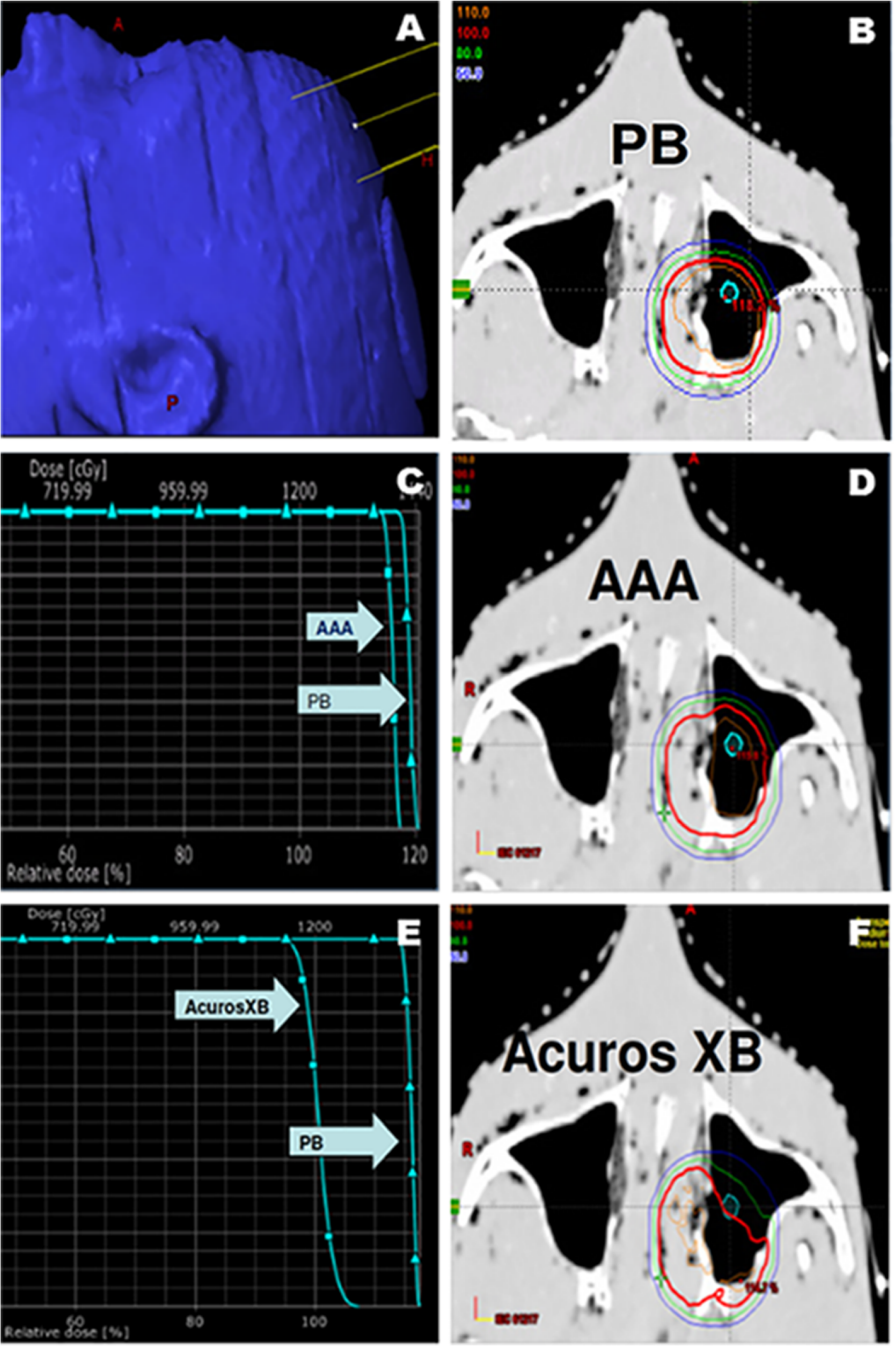
**a** Rando Man® phantom irradiated with an anterior oblique beam through the sinus cavity. **b** Predicted isodose distribution through sinus cavity by PB algorithm. **c** Dose Volume Histogram (DVH) comparing dose to structure in air for PB versus AAA. **d** Predicted isodose distribution by AAA algorithm. **e** DVH comparing dose to structure in air by PB versus AXB. **f** Predicted isodose distribution by AXB algorithm

**Fig 3:**
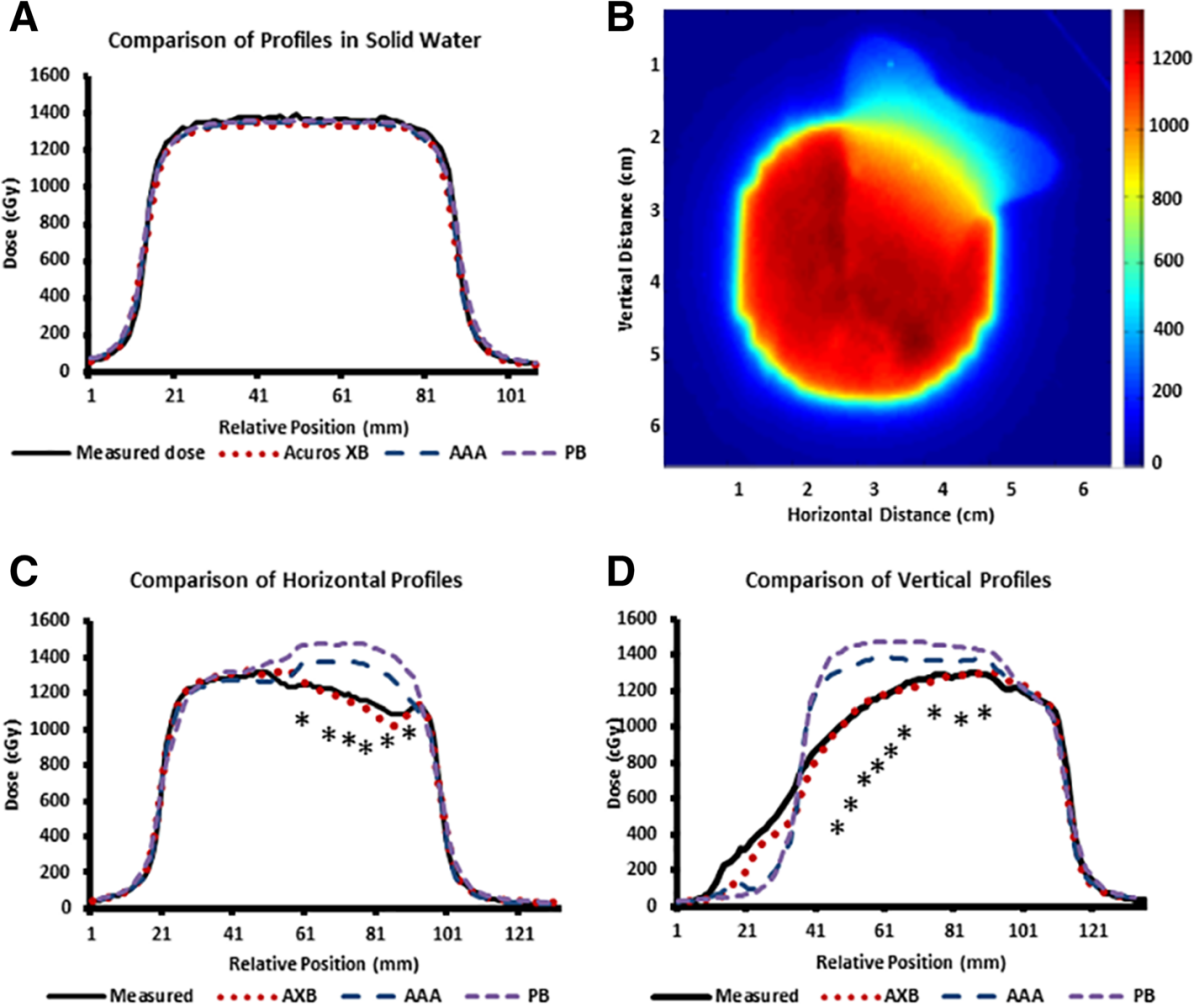
**a** Dose profile in solid water phantom compared with predicted dose from AXB, AAA, and PB. **b** Film exposure after irradiation of sinus target from Fig. 2. **c** Horizontal dose profile from Fig. 3b compared with predicted dose from AXB, AAA, and PB. **d** Vertical dose profile from Fig. 3b compared with predicted dose from AXB, AAA, and PB. * indicates *p* < 0.05 for all figures between AXB and both PB and AAA

Given the differences in prediction for the three algorithms found above, we sought to determine the absolute delivered dose for both uniform and heterogeneous density materials. The Rando Man® phantom from The Phantom Laboratory was used to test the dose prediction near heterogeneities [17]. The phantom was irradiated with an anterior oblique beam through the sinus cavity (Fig. 2a), with a path through several interfaces between air, soft tissue, and bone. The calculated dose in the phantom with PB, AAA, and AXB algorithms is shown in Fig. 2 (Fig. 2b-f). Within the solid water phantom, the PB, AAA, and AXB algorithms each accurately predicted the delivered dose (+/- 1 % max) (Fig. 3a). The dose measured with GAFCHROMIC® film in the Rando compared to the dose predicted by PB, AAA, and AXB algorithms (Fig. 3b). Within the Rando Man® phantom, the PB and AAA algorithms predicted an overestimation of the dose delivered in the bone-tissue-air interface of the sinus (+17 %), while the AXB algorithm closely predicted the actual dose delivered through the inhomogeneous tissue (+/- 1 % max, P <0.05) (Fig. 3c,d).

## Discussion

In this study of 15 patients with tumors of the pituitary or cavernous sinus, we investigated whether frameless IM-SRS planning with uniform tissue density corrections results in dose inaccuracy compared to algorithms accounting for tissue heterogeneity. We found that, in general, there was no difference in target coverage for cases that were in the cavernous sinus or pituitary fossa regardless of algorithm used. However, the maximum brainstem dose was higher for PB compared to AAA and AXB, suggesting that uniform density calculations could alter dose to critical OAR. Based on these initial findings, we sought to identify the clinical situations where heterogeneity corrections were essential.

Since the dose limits to the optic nerves and chiasm are well-established for single fraction radiosurgery, we also investigated the relationship between the location of the tumor and dose limits in single fraction cases. There were 10 patients with a prescription dose ranging from 15-21Gy and a mean of 17.3Gy. PB predicted a statistically significant higher maximum dose than either AAA or AXB to the chiasm and the optic nerves bilaterally. Therefore, the use of a uniform density algorithm in these cases predicted a higher risk of OAR toxicity.

Finally, we investigated the influence of density correction algorithms when IMRT optimization would be most complex, specifically, when OAR and PTV overlap. All three algorithms did not influence optimization to protect OAR as there was significantly lower minimum dose to the PTV with OAR overlap versus cases without OAR overlap. However, PB predicted a Dmax above 8Gy to the optic nerves for similar target coverage compared with either heterogeneity-corrected algorithm. The AAA dose to the optic nerves was near 8Gy at 7.69Gy for the overlap cases while AXB was less at 6.6Gy. We observed across all three analyses that PB consistently predicted a higher dose to OAR for similar target coverage. Given that clinical toxicity ultimately occurs as a result of deposited dose, we investigated which of the algorithms accurately predicted dose in uniform and hetereogeneous density phantoms.

After irradiation of a solid water or rando phantom, we measured the deposited dose using film. All three algorithms accurately predicted the dose in the solid water phantom. The rando phantom exemplifies a unique model of bone-tissue-air interface of the sinus and was used to compare the predicted dose as discerned by each algorithm to the measured dose for a target near several interfaces across varying densities. Within the rando phantom, the PB and AAA algorithms predicted an overestimation of the dose delivered in the bone-tissue-air interface of the sinus, while the AXB algorithm closely predicted the actual dose delivered through the inhomogeneous tissue. These results taken together suggest that AXB would most accurately predict dose deposition in patients, and PB would falsely predict toxicity due to overestimation of dose to OAR. This work expands upon previous observations made using density-correction in dose calculation.

PB algorithms employ pencil beam kernels to predict the distribution of dose from a very narrow beam or beamlet. A treated beam is divided into several such narrow beamlets and the calculated dose to a voxel is the sum of the dose delivered per beamlet. The kernel defines the spatial distribution of energy following interactions in water. To account for heterogeneities, the Brainlab PB algorithm adjusts the depth used for dose calculation to a radiological path length, or a depth of water equivalent to the depth in tissue when accounting for differences in attenuation coefficients. There is no such radiological path length correction applied to the spatial parameters of the kernel. When heterogeneities lie in the volume of tissue immediately surrounding the voxel of interest, the effect of such heterogeneities is not considered. In the case of AAA, the option to use heterogeneous calculations includes a scaling of the scatter kernels according to the radiological depth. This accounts for effects of the heterogeneities in the area surrounding a voxel of interest.

The AAA [10, 11, 18] was implemented in the Eclipse treatment planning system (Varian Medical Systems, Palo Alto, CA) to replace the PB algorithm [8, 9] for calculating dose distributions for photon beams. The goal of the AAA was to enhance the accuracy of dose calculations in heterogeneous media. The newer AXB represents a photon dose calculation algorithm which is implemented in the Eclipse treatment planning system [12]. AXB appears to offer a valid and accurate alternative to Monte Carlo calculations for heterogeneity calculations [12].

Algorithms which account for tissue heterogeneity do so with varying degrees of complexity and accuracy. PB has been the longstanding algorithm of choice in radiosurgery applications with a very simple method of correcting for differences in electron density. The simplified method allows for fast calculation times, and in many intracranial applications produces congruent results in comparison to the more complex and rigorous heterogeneity corrections in algorithms like AAA and AXB. However, in areas of complex geometric heterogeneities, the predictions of the respective algorithms diverge.

Radiosurgery dose constraints for OAR are based on clinically observed adverse events, which often take months or years to become apparent. The dose for the optic structures of 8Gy appears to be conservative and based on CT-only based planning. Incorporation of MRI guidance for SRS resulted in a tolerance of 10Gy, but only with cases without tumor overlapping the PTV [19]. Therefore, as technology of SRS has evolved, so have the OAR dose tolerances. However, due to the timing of the late effects, there is a lag of understanding of the clinical implications of the implemented technology. We have observed that for the same PTV dose, AAA and AXB both predict lower doses to the optic nerves and chiasm (Fig. 1b and c) ranging from 10 to 13 %. Our phantom measurements confirm AXB models the dose to these tissues more accurately than PB. This may mean that the actual tolerance of the optic structures is 10 % less than the 10Gy observed above with PB algorithms, at 9Gy. Additional follow-up of patients treated with AXB is necessary to confirm.

The present work relied on initial optimization of fluence of beamlets using a simplified DVO algorithm for calculation speed. Subsequent dose calculation was done with PB, AAA, or AXB on the DVO fluence. Given the disparity of calculated and measured dose from PB, AAA, and AXB, we conducted a preliminary analysis investigating heterogeneity-corrected optimization of four uniform patients each with PTV overlapping the right optic nerve and a prescription dose of 15Gy. AAA and AXB were both used for the intermediate dose calculation. Automated optimization was performed which included optimization with DVO, dose calculation with the specified volume dose calculation algorithm, and repeated optimization utilizing the dose calculation information as input for the subsequent optimization. Maximum dose to the optic nerves for PB optimized and calculated plans was 853 cGy compared to 663 with AAA optimized and calculated plans (*p* = 0.02). The maximum optic nerve dose with AXB optimized and calculated plans was 685 cGy (*p* = 0.02). Although statistically significant, these results are not conclusive but support further investigation based on heterogeneity-corrected optimization.

Several studies have investigated the impact of the AXB algorithm for intensity-modulated radiation therapy, specifically, in nasopharyngeal carcinomas [20], lung carcinoma [21], and the experimental use of AXB with a head and neck phantom [22]. Using both AXB and AAA algorithms, Kan et al. investigated the dose distributions to numerous PTVs with different prescribed doses and critical organs [20]. The PTVs were divided into bone, air, and tissue components. They determined that AXB is recommended for IMRT and RapidArc planning for patients with nasopharyngeal carcinomas, which have a similar location to the cases in the present study.

Han et al. studied how AXB functioned for both intensity-modulated radiation therapy IMRT and volumetric-modulated arc therapy (VMAT) [22]. They used a head and neck phantom to calculate dose distributions for AXB and AAA. Han et al. determined that there was a good agreement between measured doses and those calculated with AXB and AAA [22]. AXB findings were equal to or better than those discerned with film measurements for IMRT and VMAT plans. Furthermore, they demonstrated that the AXB calculation time was four times shorter than AAA for VMAT, which may be important during optimization of cases with extensive PTV and OAR overlap.

Han and Followill reported the role of the AXB algorithm for heterogeneous dose calculation in lung cancer [21]. Utilizing a thorax phantom, they showed that AXB was accurate for dose calculation in lung cancer for both IMRT and VMAT. In addition, they determined that AXB may improve accuracy and reduce computation time in lung VMAT [21]. The present study represents the first in the literature that explored the heterogeneity correction for frameless IM-SRS in pituitary and cavernous sinus tumors. The findings of the present study reflect those of previous works [20–22] in that AXB is beneficial in heterogeneous dose corrections.

## Conclusions

Patients undergoing frameless SRS benefit from heterogeneity corrected dose plans when the lesion lies in areas of widely varying tissue density and near critical normal structures. A uniform density algorithm overestimates the dose to OAR compared with heterogeneity algorithms for patients with OAR and PTV overlap. Furthermore, film dosimetry confirms the AcurosXB dose calculation algorithm more accurately predicts deposited dose though tissues of varying densities than PB or AAA dose calculation algorithms. Our results suggest that patients undergoing radiosurgery for base of skull lesions may benefit from AXB calculations when OAR and PTV overlap during the planning process. However, there are other algorithms including collapsed cone which was not studied here. Advanced heterogeneity corrections should be validated with phantom measurement to simulate a given clinical scenario.
